# Supplementary material for: Correlates of protection against African swine fever virus identified by a systems immunology approach
Source: eLife. 2026 Jun 29;14:RP107579. doi: 10.7554/eLife.107579 (PMC13313700; doi:10.7554/eLife.107579)
Supplement: Peer review history

# Editor report: eLife Assessment

**DOI:** 10.7554/eLife.107579.3.sa0

**Authors:** Florent Ginhoux

**Affiliations:** Singapore Immunology Network Singapore

**Keywords:** Solid, Valuable

This study provides **valuable** findings regarding potential correlates of protection against the African swine fever virus. The evidence supporting the claims is **solid**, and the results are highly relevant to the field. Further analysis using larger number of animals and other virus strains will help validate the importance of these findings and assess the relevance of the immune parameters associated with protection. The work will be of broad interest to veterinary immunologists, and particularly those working on African swine fever.

# Referee report: Reviewer #1 (Public review)

**DOI:** 10.7554/eLife.107579.3.sa1

The study by Lotonin et al. investigates correlates of protection against African swine fever virus (ASFV) infection. The study is based on a comprehensive work, including the measurement of immune parameters using complementary methodologies. An important aspect of the work is the temporal analysis of the immune events, allowing to capture the dynamics of the immune responses induced after infection. Also, the work compares responses induced in farm and SPF pigs, showing the later an enhanced capacity to induce a protective immunity. Overall, the results obtained are interesting and relevant for the field. The findings described in the study further validate work form previous studies (critical role of virus-specific T cell responses), and provide new evidence on the importance of a balanced innate immune response during the immunization process. This information increases our knowledge on basic ASF immunology, one of the important gaps in ASF research that needs to be addressed for a more rational design of effective vaccines. As discussed in the manuscript, the results provide targets which can be further validated in other models, such as immunization using live attenuated vaccines.

Overall the conclusions of the work are well supported by the results, and most of the issues mentioned during the review have been properly addressed during the revision, improving the quality of the final manuscript. While some limitations remain, I consider that they do not invalidate the results obtained and are well discussed by the authors.

The study is highly relevant for the field, representing a step forward in our understanding of ASF protective immunity, providing immune targets to be further explored in other models and during vaccine development.

# Referee report: Reviewer #2 (Public review)

**DOI:** 10.7554/eLife.107579.3.sa2

Summary:

In the current study the authors attempt to identify correlates of protection for improved outcomes following re-challenge with ASFV. An advantage is the study design which compares the responses to a vaccine like mild challenge and during a virulent challenge months later. It is a fairly thorough description of the immune status of animals in terms of T cell responses, antibody responses, cytokines and transcriptional responses and the methods appear largely standard. The comparison between SPF and farm animals is interesting and probably useful for the field in that it suggests that SPF conditions might not fully recapitulate immune protection in the real world. I thought some of the conclusions were over-stated and there are several locations where the data could be presented more clearly.

Strengths:

The study is fairly comprehensive in the depth of immune read-outs interrogated. The potential pathways are systematically explored. Comparison of farm animals and SPF animals gives insights into how baseline immune function can differ based on hygiene, which would also likely inform interpretation of vaccination studies going forward.

Weaknesses:

There are limited numbers of animals assessed.

Comments on revisions:

The authors mostly addressed my comments to the previous version. However, in the discussion they added comments relating to and an interpretation based on their own unpublished data and I think that those statements should be removed because the data are not included in this publication and cannot be cited.

# Author comment: Author response

**DOI:** 10.7554/eLife.107579.3.sa3

**Authors:** Kirill Lotonin, Francisco Brito, Kemal Mehinagic, Obdulio García-Nicolás, Matthias Liniger, Noelle Donzé, Sylvie Python, Stephanie Talker, Tosca Ploegaert, Nicolas Ruggli, Charaf Benarafa, Artur Summerfield

**Affiliations:** Institute of Virology and Immunology Mittelhäusern Switzerland; Institute of Virology and Immunology Mittelhäusern Switzerland; Institute of Virology and Immunology Mittelhäusern Switzerland; Institute of Virology and Immunology Mittelhäusern Switzerland; Institute of Virology and Immunology Mittelhäusern Switzerland; Institute of Virology and Immunology Mittelhäusern Switzerland; Institute of Virology and Immunology Mittelhäusern Switzerland; Institute of Virology and Immunology Bern Switzerland; Wageningen University and Research Lelystad Netherlands; Institute of Virology and Immunology Mittelhäusern Switzerland; Institute of Virology and Immunology Mittelhäusern Switzerland; Institute of Virology and Immunology Mittelhäusern Switzerland

The following is the authors’ response to the original reviews.

**Public Reviews:**

**Reviewer #1 (Public review):**

The study by Lotonin et al. investigates correlates of protection against African swine fever virus (ASFV) infection. The study is based on a comprehensive work, including the measurement of immune parameters using complementary methodologies. An important aspect of the work is the temporal analysis of the immune events, allowing for the capture of the dynamics of the immune responses induced after infection. Also, the work compares responses induced in farm and SPF pigs, showing the latter an enhanced capacity to induce a protective immunity. Overall, the results obtained are interesting and relevant for the field. The findings described in the study further validate work from previous studies (critical role of virus-specific T cell responses) and provide new evidence on the importance of a balanced innate immune response during the immunization process. This information increases our knowledge on basic ASF immunology, one of the important gaps in ASF research that needs to be addressed for a more rational design of effective vaccines. Further studies will be required to corroborate that the results obtained based on the immunization of pigs by a not completely attenuated virus strain are also valid in other models, such as immunization using live attenuated vaccines.

While overall the conclusions of the work are well supported by the results, I consider that the following issues should be addressed to improve the interpretation of the results:

We thank Reviewer #1 for their thoughtful and constructive feedback, which significantly contributed to improving the clarity and quality of our manuscript. Below, we respond to each of the reviewer’s comments and describe the revisions that were incorporated.

(1) An important issue in the study is the characterization of the infection outcome observed upon Estonia 2014 inoculation. Infected pigs show a long period of viremia, which is not linked to clinical signs. Indeed, animals are recovered by 20 days post-infection (dpi), but virus levels in blood remain high until 141 dpi. This is uncommon for ASF acute infections and rather indicates a potential induction of a chronic infection. Have the authors analysed this possibility deeply? Are there lesions indicative of chronic ASF in infected pigs at 17 dpi (when they have sacrificed some animals) or, more importantly, at later time points? Does the virus persist in some tissues at late time points, once clinical signs are not observed? Has all this been tested in previous studies?

Tissue samples were tested for viral loads only at 17 dpi during the immunization phase, and long-term persistence of the virus in tissues has not been assessed in our previous studies. At 17 dpi, lesions were most prominently observed in the lymph nodes of both farm and SPF pigs. In a previous study using the Estonia 2014 strain (doi: 10.1371/journal.ppat.1010522), organs were analyzed at 28 dpi, and no pathological signs were detected. This finding calls into question the likelihood of chronic infection being induced by this strain.

(2) Virus loads post-Estonia infection significantly differ from whole blood and serum (Figure 1C), while they are very similar in the same samples post-challenge. Have the authors validated these results using methods to quantify infectious particles, such as Hemadsorption or Immunoperoxidase assays? This is important, since it would determine the duration of virus replication post-Estonia inoculation, which is a very relevant parameter of the model.

We did not perform virus titration but instead used qPCR as a sensitive and standardized method to assess viral genome loads. Although qPCR does not distinguish between infectious and non-infectious virus, it provides a reliable proxy for relative viral replication and clearance dynamics in this model. Unfortunately, no sample material remains from this experiment, but we agree that subsequent studies employing infectious virus quantification would be valuable for further refining our understanding of viral persistence and replication following Estonia 2014 infection.

(3) Related to the previous points, do the authors consider it expected that the induction of immunosuppressive mechanisms during such a prolonged virus persistence, as described in humans and mouse models? Have the authors analysed the presence of immunosuppressive mechanisms during the virus persistence phase (IL10, myeloid-derived suppressor cells)? Have the authors used T cell exhausting markers to immunophenotype ASFV Estonia-induced T cells?

We agree with the reviewer that the lack of long-term protection can be linked to immunosuppressive mechanisms, as demonstrated for genotype I strains (doi: 10.1128/JVI.00350-20). The proposed markers were not analyzed in this study but represent important targets for future investigation. We addressed this point in the discussion.

(4) A broader analysis of inflammatory mediators during the persistence phase would also be very informative. Is the presence of high VLs at late time points linked to a systemic inflammatory response? For instance, levels of IFNa are still higher at 11 dpi than at baseline, but they are not analysed at later time points.

While IFN-α levels remain elevated at 11 dpi, this response is typically transient in ASFV infection and likely not linked to persistent viremia. We agree that analyzing additional inflammatory markers at later time points would be valuable, and future studies should be designed to further understand viral persistence.

(5) The authors observed a correlation between IL1b in serum before challenge and protection. The authors also nicely discuss the potential role of this cytokine in promoting memory CD4 T cell functionality, as demonstrated in mice previously. However, the cells producing IL1b before ASFV challenge are not identified. Might it be linked to virus persistence in some organs? This important issue should be discussed in the manuscript.

We agree that identifying the cellular source of IL-1β prior to challenge is important, and this should be addressed in subsequent studies. We included a discussion on the potential link between elevated IL-1β levels and virus persistence in certain organs.

(6) The lack of non-immunized controls during the challenge makes the interpretation of the results difficult. Has this challenge dose been previously tested in pigs of the age to demonstrate its 100% lethality? Can the low percentage of protected farm pigs be due to a modulation of memory T and B cell development by the persistence of the virus, or might it be related to the duration of the immunity, which in this model is tested at a very late time point? Related to this, how has the challenge day been selected? Have the authors analysed ASFV Estonia-induced immune responses over time to select it?

In our previous study, intramuscular infection with ~3–6 × 10^2^ TCID_50_/mL led to 100% lethality (doi: 10.1371/journal.ppat.1010522), which is notably lower than the dose used in the present study, although the route here was oronasal. The modulation of memory responses could be more thoroughly assessed in future studies using exhaustion markers. The challenge time point was selected based on the clearance of the virus from blood and serum. We agree that the lack of protection in some animals is puzzling and warrants further investigation, particularly to assess the role of immune duration, potential T cell exhaustion caused by viral persistence, or other immunological factors that may influence protection. Based on our experience, vaccine virus persistence alone does not sufficiently explain the lack-of-protection phenomenon. We incorporated these important aspects into the revised discussion.

(7) Also, non-immunized controls at 0 dpc would help in the interpretation of the results from Figure 2C. Do the authors consider that the pig's age might influence the immune status (cytokine levels) at the time of challenge and thus the infection outcome?

We support the view that including non-immunized controls at 0 dpc would strengthen the interpretation of cytokine dynamics and will consider this in future experimental designs. Regarding age, while all animals were within a similar age range at the time of challenge, we acknowledge that age-related differences in immune status could influence baseline cytokine levels and infection outcomes, and this is an important factor to consider.

(8) Besides anti-CD2v antibodies, anti-C-type lectin antibodies can also inhibit hemadsorption (DOI: 10.1099/jgv.0.000024). Please correct the corresponding text in the results and discussion sections related to humoral responses as correlates of protection. Also, a more extended discussion on the controversial role of neutralizing antibodies (which have not been analysed in this study), or other functional mechanisms such as ADCC against ASFV would improve the discussion.

The relevant text in the Results and Discussion sections was revised accordingly, and the discussion was extended to more thoroughly address the roles of antibodies.

**Reviewer #2 (Public review):**

Summary:

In the current study, the authors attempt to identify correlates of protection for improved outcomes following re-challenge with ASFV. An advantage is the study design, which compares the responses to a vaccine-like mild challenge and during a virulent challenge months later. It is a fairly thorough description of the immune status of animals in terms of T cell responses, antibody responses, cytokines, and transcriptional responses, and the methods appear largely standard. The comparison between SPF and farm animals is interesting and probably useful for the field in that it suggests that SPF conditions might not fully recapitulate immune protection in the real world. I thought some of the conclusions were over-stated, and there are several locations where the data could be presented more clearly.

Strengths:

The study is fairly comprehensive in the depth of immune read-outs interrogated. The potential pathways are systematically explored. Comparison of farm animals and SPF animals gives insights into how baseline immune function can differ based on hygiene, which would also likely inform interpretation of vaccination studies going forward.

Weaknesses:

Some of the conclusions are over-interpreted and should be more robustly shown or toned down. There are also some issues with data presentation that need to be resolved and data that aren't provided that should be, like flow cytometry plots.

We appreciate the feedback from the Reviewer #2 and acknowledge the concerns raised regarding data presentation. In the revised manuscript, we clarified our conclusions where needed and ensured that interpretations were better aligned with the data shown.

**Recommendations for the authors:**

**Reviewer #1 (Recommendations for the authors):**

(1) In the Introduction, more details on the experimental model would be appreciated. A short summary of findings obtained with this model in previous works from the authors would help to better understand the context of the study.

Basic information on the model was added in the Introduction section of the revised manuscript.

(2) In Figure 1, the addition of more time points on the x-axes would help the interpretation of the figures.

We agree and have added extra time points to the x-axes.

(3) To better understand the results in Figure 2A, a figure showing cytokine levels post-Estonia infection of only challenged pigs would help, indicating protected and non-protected animals as in Figure 2C. This figure would be better linked to the corresponding dot plot (Figure 2B).

Our statistical analyses in Figure 2A are based on using both challenged and non-challenged pigs to assess differences between SPF and farm pigs. We prefer not to remove the non-challenged pigs in order to avoid losing statistical power. Moreover, even when non-challenged and challenged pigs are displayed in the plots, upregulation of IFN-α and IL-8 can be visualized and remains consistent with the positive and negative correlates of protection shown in Figure 2C.

(4) Dark red colour associated with SPF non-protected is difficult to differentiate from light red in some figures.

We thank the reviewer for this remark. To preserve the color scheme across the paper, we changed the circle data points to squares for the non-protected SPF pig in the most crowded figures: Figures 1–3 and Supplementary Figures 2 and 8.

(5) In Supplementary figures 12-16, grouping of the animal numbers (SPF vs farm) would facilitate the interpretation of the results.

Information on the animal numbers for each group (SPF vs. farm) has been added to the figure captions.

(6) Are the results shown in Figure 8 based on absolute scores as mentioned? Results from 0 dpc are not shown. Is that correct?

That is correct. BTM expression values are absolute and could not be normalized, as RNA was not isolated either immediately before the challenge or on day 0 post-challenge. This information is now clarified in the figure captions.

**Reviewer #2 (Recommendations for the authors):**

(1) The authors use the words "predicted" and "predicts" although they haven't used any methods to show that this is true, such as a multivariate analysis. I don't think correlation coefficients are sufficient to indicate prediction. This needs to be fixed.

We agree with this and have made changes in the text to avoid this impression.

(2) "Lower baseline immune activation was linked to increased protective immunity." Presumably, the authors mean prior to challenge, not prior to "vaccination"?

In this sentence written in the Abstract, we refer to baseline immune activation in the steady state, i.e., prior to any infection, as demonstrated in a previous study by Radulovic et al. (2022). The sentence was adapted accordingly. This concept is further explored in the Discussion section.

(3) The abstract mentioned the comparison between farm and SPF pigs, but didn't provide any context for those findings. It could be added here.

In the new version, we have added information on this model in the Introduction section.

(4) Figure legends need N to be indicated. For example, the viral load figures don't appear to be representative of all 9 or 5 animals. Is there a reason why not all were challenged, and how were those 5 challenged selected?

Numbers of animals in each group were added to the figure captions. We have also provided details regarding the animals sacrificed at different time points of the experiment in the ‘Animal experiment’ section of the Methods.

(5) 1A doesn't have a legend to indicate whether dark or light color indicates sampling.

Fair point. We have added the information to the figure.

(6) For Figure 3C, it's not clear how the correlation is presented. The legend indicates in writing that the color indicates the outcome it correlates with, but the legend suggests that it is r.

The method of presenting correlation data is consistent across all figures, including Figure 3C. The color reflects the direction and strength of the correlation, corresponding to the r coefficient obtained from correlating immunological parameters with clinical scores. We have clarified this description in the figure caption to improve readability.

(7) For some of the correlation data in 2D and 3C, it would be nice to provide the plots in the supplemental. Also, are there enough data points for a robust interpretation of correlation curves?

We agree that providing the plots will improve clarity and have included them in the supplementary material. While we acknowledge that the number of data points is modest, we believe it is sufficient to support a robust interpretation of the correlation curves. Corresponding p-value cutoffs are noted in the figure captions.

(8) The figure 2C method of indicating significance is confusing. There must be a clearer way to present this figure.

Analyzing statistical significance for the dataset shown in Figure 2C is challenging due to the small number of animals. We carefully considered alternative ways of presenting statistical significance, however, given the limited group sizes, we believe that the current approach provides the most transparent and informative representation of the data.

For clarity, we divided the animals into SPF and farm groups, as well as into protected (4 SPF, 2 farm pigs) and non-protected (1 SPF, 3 farm pigs) categories, and performed both group-based (unpaired t-test) and time-based (mixed-effects analysis) comparisons. All significant differences were added to the plots so that readers could directly visualize the observed trends and compare them with the correlation analysis presented in Figure 2D.

(9) Please note that "viremia" means the presence of a virus specifically in the blood. Other descriptions of viral load should be used if this was not measured.

We have clarified this in the text. When referring to organs, we use the term “viral loads.”

(10) The way of putting a square around boxes that are significant can be misleading when a box is surrounded by other significant comparisons. Like for Figure 6B - probably all of these are really significant, but I can't tell for sure.

Good point. We changed rectangles to circles for better readability of the figures.

(11) There is a potential argument that these correlates of protection might only be valid for this specific vaccine. It should be noted that comparisons of multiple vaccines would be needed before assuming the correlates are broadly relevant.

We agree with this statement and address it in the Discussion section.

(12) For the circled pathways in Figure 9, it is not clear from the diagram if there is a directionality to the involvement of those pathways. Modulated or induced?

When discussing pathways identified by transcriptome analysis, we are always referring to their induction, as this is based on the normalized enrichment score (NES). We have now specified this in the figure caption.

(13) The authors speculate about NK cells, but this is based on transcriptional pathways identified and the literature. Is there any indication from the flow cytometry data whether activated NK cells versus NKT cells are associated with protection? Also, the memory phenotype of those cells?

Regarding NK cells, the BTM analysis was corroborated by the flow cytometry data shown in Supplementary Figure 8. NK cells were defined as CD3^-^CD8α^+^. Specific markers to distinguish NKT cells or to assess memory phenotypes were not included in our panel.

(14) In the discussion, "Our study demonstrates that T cell activation represents a robust correlate of protection against ASFV" doesn't indicate whether they mean after vaccination or after challenge. Re-using the same time points throughout the manuscript compounds this confusion.

In this case, we mean that T cell activation upon immunization/vaccination and challenge correlates with protection. This information has been added to the sentence. Although some time points overlap between the immunization and challenge phases, we consistently use “dpi” and “dpc” to clearly distinguish them.

(15) Flow cytometry gating strategies should be provided in the supplemental, particularly since this species is less frequently studied using flow cytometry; it would be helpful to understand gating and expression levels of key markers.

We have provided the gating strategy in Supplementary Figure 7, which is also referenced in the “Flow cytometry and hematology analysis” section of the Methods.

(16) Some of the discussion is a bit long and repetitive - e.g. the parts on antibodies and the last paragraph with multiple other parts of the discussion and manuscript.

While we agree that some sections are extensive, we think that this level of detail is necessary to integrate the different datasets and to place our findings in the context of previous literature.
